# Determining the metabolic profile measured using ultra-high pressure liquid chromatography associated with Beta Amyloid burden in the brain in individuals with mild cognitive impairment

**DOI:** 10.21203/rs.3.rs-10065374/v1

**Published:** 2026-07-31

**Authors:** Marjan Falahati, Elham Ramezannejad, Yosra Vaez-Gharamaleki, Parastoo Radnia, Saman Shahba, Pegah Rasoulian, Sara Rezaei, Negin Dasmeh, Maryam Bemanalizadeh

**Affiliations:** Kermanshah University of Medical Science; Isfahan University of Medical Science; Tabriz University of Medical Sciences; Tabriz University of Medical Sciences; Mashhad University of Medical Science; Tehran University of Medical Sciences; Islamic Azad University; Iran University of Medical Sciences; Isfahan University of Medical Sciences

**Keywords:** Alzheimer’s disease, Amyloid PET, ultra-high pressure liquid chromatography, Dementia, Biomarkers, Metabolic networks

## Abstract

As the world’s population ages, Alzheimer’s disease (AD), a progressive neurodegenerative disorder, is becoming more common. The current diagnoses for AD are not precise enough to identify the disorder when it is possible to cure. An early indicator of Alzheimer’s is the assessment of beta-amyloid by positron emission tomography (PET) score, a substantial decrease in the brain’s metabolomics. Furthermore, Metabolomics can be detected by UPLC (ultra-high pressure liquid chromatography). This study investigates the link between metabolic profiles and beta-amyloid load in AD in order to shed light on possible biomarkers for disease progression.

The data from 59 people who have been diagnosed with moderate cognitive impairment (MCI) is extracted from the Alzheimer’s Disease Neuroimaging Initiative (ADNI).

This study uses ultra-high-performance liquid chromatography (UPLC) to investigate the correlation between baseline metabolic data and changes in amyloid PET scores. Seven metabolites, including DOPA, Methionine Sulfoxide (Met.SO), Tryptophan Betaine, choline, leucine, valine, and FA (18:2), seem to have a substantial connection with amyloid PET scores. These can be used as early indications of AD progression, especially Met.SO is particularly relevant when considering time interaction. Understanding the metabolic alterations associated with amyloid load can pave the way for future research incorporating metabolites into a therapeutic treatment that can be identified as crucial to the illness’s progression.

## 1. Introduction

Alzheimer’s dementia (AD) comprises a spectrum of neurodegenerative disorders characterized by memory impairment and progressive decline in cognitive function. This debilitating condition results in challenges in daily tasks necessary for independent living (Eschweiler, Leyhe, Klöppel, & Hüll, 2010). Amyloid-β (Aβ) and tau accumulations are strongly linked to AD and their impact on cognitive function and neurodegeneration in humans (Jagust, 2018).

Recent breakthroughs in neuroimaging have improved the non-invasive observation and quantification of Aβ burden in living individuals using positron emission tomography (PET) scans with radio ligands mainly designed to attach to amyloid aggregates. Among these radio ligands, [(^11^C]SB-13, [^11^C]PIB, and [^18^F]FDDNP have received attention due to their great sensitivity and specificity in detecting Aβ plaques. PET imaging enables researchers and physicians to visualize and quantify the degree of Aβ accumulation in various brain regions. This data is critical in understanding disease development, investigating regional variations in amyloid burden, and detecting potential links to cognitive decline and other biomarkers (Quigley, Colloby, & O’Brien, 2011).

PET imaging’s capacity to identify and quantify Aβ load has significant clinical implications. Early and precise detection of AD is critical for timely intervention, and PET scans offer a promising path for early detection before significant cognitive impairment arises. Longitudinal PET imaging investigations can also shed light on the temporal dynamics of Aβ accumulation, allowing for a better understanding of its involvement in disease development (Chételat et al., 2020).

Studies reveal that the majority of patients remain undiagnosed for years after the onset of AD pathogenesis, predominantly due to a gap between the deposition of Aβ followed by the formation of plaques in various regions of the brain and the emergence of cognitive deficit symptoms (Liss et al., 2021; Schilling et al., 2016). More recent diagnostic criteria presented by the National Institute on Aging Alzheimer’s Association (NIA-AA) utilize biomarkers along with neuropsychological assessments and neuroimaging techniques (Counts, Ikonomovic, Mercado, Vega, & Mufson, 2017). Numerous studies have indicated that biomarkers can be more effective in diagnosing AD when used in conjunction with traditional methods (Bjerke & Engelborghs, 2018; Graff-Radford et al., 2021; Khan, Barve, & Kumar, 2020). However, practically, the prevalent diagnostic approach involves performing clinical neuropsychological testing combined with the utilization of advanced neuroimaging techniques (Bjerke & Engelborghs, 2018).

Early detection of AD can provide patients with the necessary treatment to prevent the disease from progressing to more severe stages. A variety of biomarkers have been identified as essential for discriminating between different phenotypes of atypical AD, detecting dementia in its early stages, and understanding the underlying etiology of decreased cognitive abilities as well as the severity of AD progression (Bjerke & Engelborghs, 2018; Counts et al., 2017; Graff-Radford et al., 2021).

Previous studies have shown that some metabolites are involved in AD. Metabolites are biochemical products of biological pathways, such as amino acids and nucleic acids. Leucine (Leu), isoleucine (Ile), and valine (Val) are three types of essential amino acids in Branched-chain amino acids (BCAAs). Some studies have shown a relationship between amino acid metabolites and AD. The study showed a decrease in valine in cerebrospinal fluid and plasma in patients with AD (Basun et al., 1990).

In a longitudinal study on mice, they found that the plasma level of valine in AD mice was reduced. Another study showed that cognitive decline is associated with low levels of valine in AD (J. B. Toledo et al., 2017). Therefore, a decrease in the level of BCAAs might be related to AD (Green et al., 2023). Another study shows the relationship between several classes of metabolites and imaging markers, including lipids and specific amino acids, with neurodegeneration (de Leeuw et al., 2021; Kim et al., 2019; Nho et al., 2021; Proitsi et al., 2017; Xu et al., 2020). In another research, the use of the system approach to examine the correlation of metabolites with cognitive function in late middle age in the Medical Research Council National Survey of Health and Development (NSHD) showed that a group of metabolites was identified, including acylcarnitines, modified nucleotides and amino acids, vitamins, and sphingolipids (Stafford et al., 2013).

In addition, evidence points to a link between AD and vitamin B12 (Lauer et al., 2022). The investigations showed the biochemical pathway of AD that was influenced by vitamin B12 at the molecular level and in animal studies via effecting on Aβ generation by β- and γ-secretase cleavage and vitamin B12-dependent alterations of the homocysteine cycle and DNA methylation of BACE1 and PSEN1 promoters (Lauer et al., 2022). Along with other vitamin B families, vitamin B12 has beneficial effects the cognitive function, brain inflammation and atrophy in older people without cognitive decline or patients with mild cognitive decline. The plasma vitamin B12 levels were reduced in patients with cognitive decline compared to healthy individuals(Lauer et al., 2022).

We investigated the association between metabolites profile using Ultra-high-performance liquid chromatography (UPLC) and whole brain Aβ burden using PET scan as well as cognitive impairment. We investigated the potential associations to outline key blood metabolic factors in AD diagnosis and follow-up.

## 2. Methods

### 2.1 Participants

Data used in this research were obtained from the Alzheimer’s Disease Neuroimaging Initiative (ADNI). ADNI is a global research study that has been running since 2004, and the primary goal of ADNI has been to test whether serial magnetic resonance imaging (MRI), positron emission tomography (PET), other biological markers, and clinical and neuropsychological assessment can be combined to measure the progression of mild cognitive impairment (MCI) and early AD. Comprehensive information about the ADNI project can be found on the official website (adni.loni.usc.edu).

### 2.2 PET data acquisition

Mean tracer uptake in the cerebellar grey and white matter was computed and used as a reference to generate whole-brain standardized uptake value ratio (SUVR) maps of florbetapir PET scans. In addition to SUVR, the dataset contains Centiloid (CL) values to measure amyloid PET uptake, focusing on the frontal, anterior, and posterior cingulate, lateral parietal, and lateral temporal regions. Age at PET imaging, sex, and educational background were included as demographic characteristics. SUVRs were gathered from multiple amyloid PET tracers. While CL values can be compared directly, SUVRs from multiple tracers are not directly comparable, so CL values were used for comparison. SUVR and CL values reflect data intensity normalized by the whole cerebellum, with a mean uptake intensity of “1.0”. The mean uptake (SUVR) within each Freesurfer Desikan-Killiany region, the cortical summary region, and five reference regions were calculated. Each amyloid PET scan was registered to the corresponding segmented/parcellated MRI, normalizing the PET scan by the entire cerebellum and the five reference regions.

### 2.3 Cognitive assessments

Cognitive evaluation was performed using the clinical Dementia Rating (CDR), Alzheimer’s Disease Assessment Scale (ADAS), and Mini-Mental State Examination (MMSE). These instruments are reliable means of evaluating cognitive and non-cognitive behavioral dysfunctions in individuals with dementia spectrum disorder. The CDR is a validated tool for use in clinical investigations on the spectrum that uses structured interviews to evaluate three areas of function (home/hobbies, community affairs, and personal care) and three domains of cognition (memory, orientation, and judgment/problem-solving). As in this study, the scores for the six domains (range from 0 to 3) assessed can be totaled (CDR-SB, or CDR sum of boxes) (Dickerson, Sperling, Hyman, Albert, & Blacker, 2007; Hughes, Berg, Danziger, Coben, & Martin, 1982; McDougall et al., 2021).

The ADAS-cog has raw values ranging from 0 to 70, where higher scores denote more severe cognitive dysfunctions. Furthermore, a 13-item version of the ADAS-cog 11 was suggested owing to some restrictions in the 11-item version. This version consists of two complementing items, a number cancellation or labyrinth problem and delayed word recall, with a score range of 0 to 8 (Battista, Salvatore, & Castiglioni, 2017; Mohs et al., 1997; Podhorna, Krahnke, Shear, E Harrison, & Initiative, 2016).

Both the 13-item and the ADAS-cog versions were used in this investigation.

Another well-known quick screening tool for clinical studies on cognitive impairment is the MMSE, whose raw values range from 0 to 30, where more severe cognitive impairment is indicated by lower scores. An accurate measure of cognitive loss in AD is the range of scores on the MMSE between 29 and 10 (Brugnolo et al., 2009).

### 2.4 Metabolites measurement

Using the Biocrates MxP Quant 500 kit, the amounts of certain metabolites were determined in 4891 human blood samples (2603 for ADNI-1 and 2288 for ADNI-GO/2). Using the methodology, we identified hundreds of metabolites from different biochemical classes which are listed in Table 1 (Table 1). To the corresponding wells, 10 μL of the blank, calibration standard, serum sample, Biocrates QC, Global Reference QC, and SPQC were introduced. At that time, the plate was dried for 30 minutes under a tender stream of nitrogen. At that point, phenyl isothiocyanate was used to derivatize the assays, and 5 mM ammonium acetic acid was eluted in methanol. For the UPLC analysis, samples were diluted with either 80:20 water-methanol (1:1) or flowing solvent (a special mixture supplied by Biocrates) for flow injection analysis (50:1).

The 152 (for ADNI-1; 150 for ADNI-GO/2) human blood samples that were examined on the first two plates were pooled into equal quantities (SPQC). The pooled test was set up in triplicate and subjected to the same analysis methods as the considered test. To vary the test’s execution throughout the test group, this test was injected into the plate three times: once before, once during, and once after the thought tests. The analyses of this sample can be used to examine possible effects on groups.

Exion AD fluid chromatography (Sciex, Framingham, MA) was used for the UPLC separation, along with a proprietary analytical column and protected column that came with the MxP Quant 500 unit (Biocrates AG, Innsbruck, Austria). A gradient from 0.2% formic acid in water to 0.2% formic acid in acetonitrile was used to separate the analytes. It took around six minutes for each test per infusion in addition to the UPLC inquiry time. A QTRAP 6500+ (Sciex) operating in the Multiple Reaction Monitoring (MRM) mode was directly fed tests for UPLC examination using two infusions, one in Electrospray Ionization Positive mode (ESI+) and one in Negative mode (ESI−).

Throughout the fitting maintenance period, MRM moves (compound-specific precursor to product ion transitions) were gathered for every analyte and inner standard. The Sciex program MultiQuantTM was used to import the UPLC-MS/MS data and perform peak integration, calibration, concentration, and computation.

Using electrospray ionization in positive mode (ESI+) on a Xevo TQ-S triple quadrupole mass spectrometer (Waters), three distinct flow injection analysis-tandem mass spectrometry (FIA-MS/MS) strategies were used to analyze acylcarnitines, monosaccharides (hexose), diglycerides, triglycerides, lysophosphatidylcholines, phosphatidylcholines, sphingomyelins, ceramides, and cholesteryl esters.

Acquity UPLC (Waters) was used for the test presentation. For every analyte and inner standard, compound-specific antecedent to item particle movements were gathered.Biocrates MetIDQTM was used to evaluate the FIA-MS/MS data.

### 2.5 Statistical analysis

Descriptive data were summarized utilizing mean and standard deviations for quantitative factors and frequencies and rates for categorical factors. F-regression highlight determination strategy from the sci-kit-learn library in Python was used to recognize the foremost pertinent metabolites for foreseeing amyloid PET score in our study. Another linear mixed effect model was utilized to analyze the longitudinal findings. For longitudinal analyses, we used the summary SUVR normalized by the composite reference region.

On amyloid PET scores, the participant was used as a random impact. The nlme bundle in R programming delicate product was utilized to fit different models considering constant variable impacts such as time, age, gender, and metabolite levels.

Demonstrate comparisons were performed utilizing the probability proportion test. Post-hoc examinations with Tukey’s alteration for numerous comparisons were conducted to look at contrasts in amyloid PET over time, focusing on noteworthy metabolite indicators. Impact sizes were surveyed utilizing negligible R-squared values determined from the linear mixed-effects model. p values of less than 0.05 were considered measurably critical.

Expecting that the R2 form is correct and all the presumptions hold, able to calculate the F measurement utilizing the standard formula, which is F = R21 - R2 x df2df. To perform an F test, able to compare this esteem with the suitable F conveyance. This comparison can be confirmed by utilizing fundamental variable-based math.

Definition df1 = k, where k represents the number of predictors. In this formula, the term df1 is referred to as the “numerator degrees of freedom” in spite of the fact that it shows up within the denomination. df2 = n–(k + 1), where n speaks to the number of observations and k speaks to the number of indicators. df2 is alluded to as the “denominator degrees of opportunity” even though it shows up within the numerator (“http://scikitlearn.org/stable/modules/generated/sklearn.feature_selection.f_regression.hml,”).

## 3. Results

This research involved 59 subjects, including MCI participants. The mean age of the subjects was 69.25 ± 6.95 years, with 42.37% (25) male and 57.63% (34) female.

Linear mixed-effects models were employed to assess the relationship between each metabolite and Aβ PET scores over time, adjusting for age, sex, and potential interactions between time and metabolite levels. The predictive value of each metabolite was evaluated using R-squared GLM, with effect sizes calculated relative to a base model without metabolite inclusion (Table 2). The table presents ten metabolites with their corresponding F-scores and P-values, highlighting their statistical significance in predicting amyloid PET scores.

In this analysis, PET score, time, age, gender, and each metabolite were run as fixed effects, and a random y-axis intercept and time slope were assigned to each subject. We ran each metabolite once with Time*metabolite interaction and once without this interaction. The results of these models are demonstrated in Table 3.

To determine whether each metabolite by itself has significant predictive value for PET scores, each model was run in an R-squaredGLM model, R^2^M and R^2^C were calculated.

Furthermore, a base linear mixed effect model without metabolite was run in this model, and effect sizes for each metabolite were calculated from the subtraction of each metabolite R-squared values from base model values (Table 4) shows the final results. This table displays the results of the linear mixed model (LMM) where selected metabolites were used as predictors of previously measured neuroimaging scores. The analysis aims to identify significant metabolites that could serve as biomarkers for Alzheimer’s disease. Dopamine, Methionine-Sulfoxide, Tryptophan betaine, Choline, Leucine, and Valine all show significant predictive values for neuroimaging scores.

**Figure 1:**
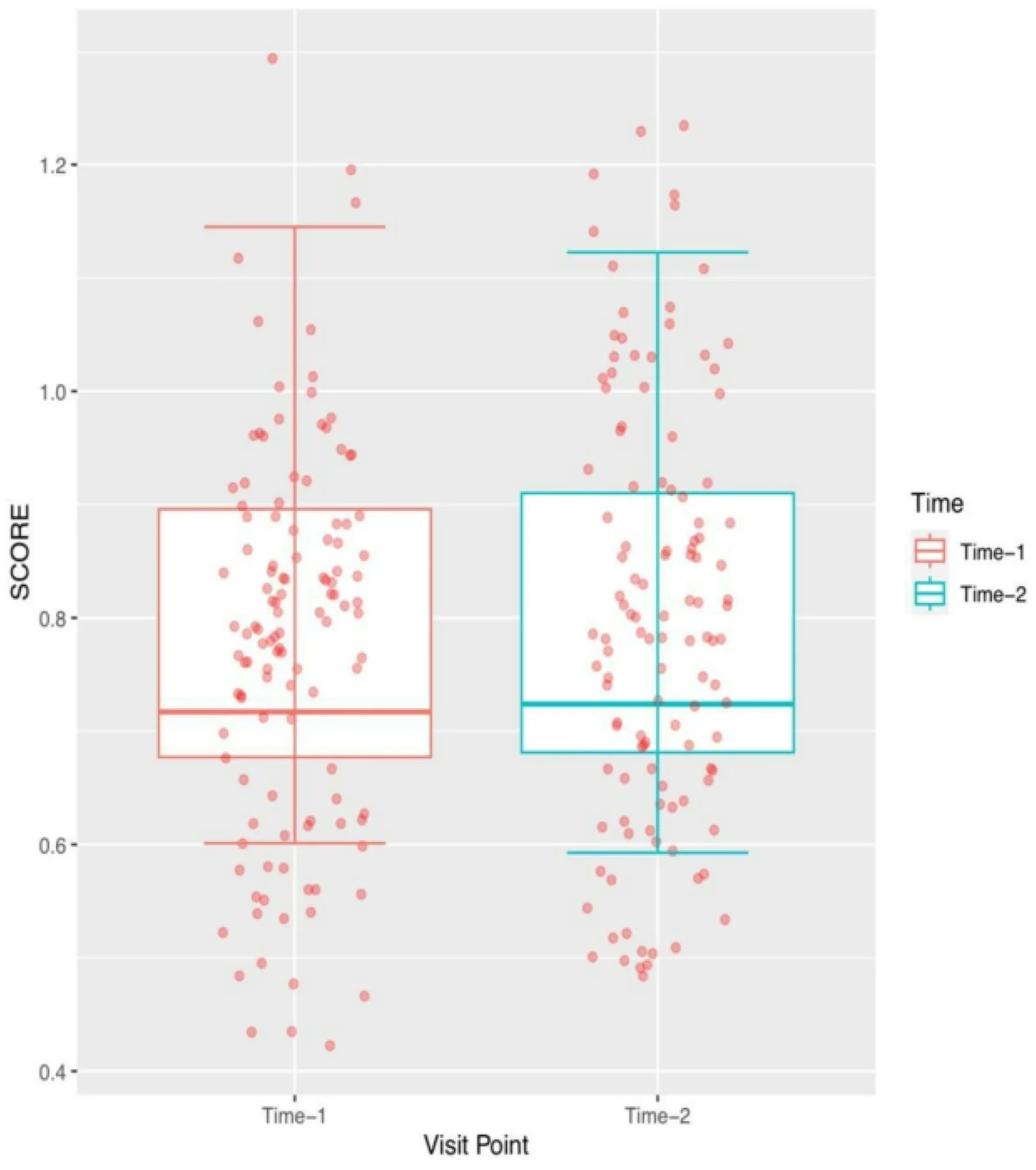
Changes in average amyloid PET scores over time in Alzheimer’s disease patients at two distinct time points.

The data was obtained through the utilization of a mixed linear effect mode. These extracted data did not include any specific visit points for the referred subjects; instead, the examination dates were grouped into two distinct visit points, namely time1 and time. Although there was a marginal increase in the scores and the average point at time 2, no significant statistical disparity was detected in the average scores between the two mentioned visit points.( Fig. 1). Notably, the primary focus of this longitudinal study is to accentuate the altering process of AB PET scores within these two-time frames rather than comparing assessed mean scores.

## 4. Discussion

We investigated the brain metabolic profiles using PET scans to discover the key biomarkers in beta-amyloid aggregation in the brain. The metabolic foundations underlying susceptibility to AD pathology and the subsequent manifestation of AD symptoms remain inadequately explained.

Additionally, the use of sensitive and selective UPLC with the highest Feature score could allow us to determine the levels of amino acids such as FA (18:2), valine, Dopamine, Leucine, Methionine Sulfoxide, Tryptophan betaine, Hypoxanthine, and Choline in the brain related to AD. Furthermore, we detected seven metabolites (DOPA, Met.SO, TrpBetaine, choline, Leu, Val, FA (18:2)) with significant predictive values without considering time interaction. However, after considering time interaction only Met.SO was statistically significant, which is persistent with results of previous targeted and nontargeted metabolomics studies in patients, particularly those composed of beta-amyloid, which is a hallmark pathological feature of AD and is believed to play a central role in the development and progression of the disease (W. C. Weng, W. Y. Huang, H. Y. Tang, M. L. Cheng, & K. H. Chen, 2019).

Moreover, a targeted metabolomics study by Weng et al. identified higher levels of Met-SO in the serums of early-stage AD patients compared to those with MCI (W.-C. Weng, W.-Y. Huang, H.-Y. Tang, M.-L. Cheng, & K.-H. Chen, 2019a)

During AD, Methionine presented in AB experiences oxidative reactions and transforms into methionine sulfoxide (Smith, Gossman, Dykstra, Gao, & Moskovitz, 2022). This oxidation is reversible through the acquisition of a methionine sulfoxide reductase (MRS) (Chandran & Binninger, 2023). However, studies suggest that the downregulation of MRS and increased rates of oxidation of amino acid residues, including methionine, are the cause for heightened levels of methionine sulfoxide (within brain cells and in serum samples in AD patients (Smith et al., 2022; W. C. Weng et al., 2019). In line with our results and evidence above, a study by Yue Deng and colleagues determined the proficiency of methionine sulfoxide as a biomarker for detecting AD in its early stages (Deng, Marsh, & Moskovitz, 2019).

It has been discovered that 10–50% of Aβ in the brain amyloid plaques of AD is made up of Met-SO (Jiang & Moskovitz, 2018).

In more severe stages of AD, the level of Met-SO should be higher since the activity of the methionine sulfoxide reductase system and other antioxidants is diminished. Our may help to explain why, in our study, early-stage AD patients had greater levels of Met-SO than MCI patients (W.-C. Weng, W.-Y. Huang, H.-Y. Tang, M.-L. Cheng, & K.-H. Chen, 2019b).

Our results indicated that dopamine, independently, has a notable predictive value, which supports previous studies highlighting lower dopamine levels in the hippocampus, frontal and prefrontal cortex in AD patients due to reduced tyrosine hydroxylase levels and deposition of AB plaques over time (Czech et al., 2012; Shaikh, Ahmad, Teoh, Kumar, & Yahaya, 2023). Furthermore, patients with AD have decreased D2-like receptors combined with D1R (Dopamine 1 Receptor) and D2R (Dopamine 2 Receptor) (Pan et al., 2019). In line with these results, Koch et al. recommended utilizing D2/D3 agonists to enhance cortical plasticity in AD (Koch et al., 2014). Dopamine plays a vital role in reducing oxidative stress and inflammation, inhibiting the formation of AB plaques and inflammatory mediators (Channer et al., 2023; Nam et al., 2018). However, our analysis suggests that the predictive value of dopamine interacting with time is not significant. This is likely due to the alternations in dopamine levels occurring later in the disease’s progression (Czech et al., 2012; Pan et al., 2019). According to a study conducted by Kristi Henjum and colleagues, our hypothesis stands validated, as they report a correlation between reduced dopamine levels and cognitive impairment rather than Alzheimer’s disease pathology itself (Henjum et al., 2022). Notably, Jarosova et al. declare lessened dopamine release in the case of AD, affecting the brain’s motor and learning functions (Jarosova, Niyangoda, Hettiarachchi, & Johnson, 2022).

Furthermore, Choline plays a critical role in various brain functions, including maintaining cell wall integrity through choline-containing phospholipids, supporting neurodevelopment throughout different life stages, and serving as a precursor for the neurotransmitter acetylcholine, which is the key to attention, learning, and memory (Francis, 2005; Peña-Bautista et al., 2020). Research has demonstrated the neuroprotective benefits of Choline (R. Bekdash, 2018; R. A. Bekdash, 2019). Another study documented amplified pro-inflammatory markers provoked by low serum choline levels(Judd et al., 2023). In the context of Alzheimer’s disease, impaired cholinergic synapses, particularly in the nucleus basalis and cell wall instability, are potential contributors to neurodegeneration (Ferreira-Vieira, Guimaraes, Silva, & Ribeiro, 2016). Additionally, the levels of Acetylcholinesterase, an enzyme responsible for breaking down acetylcholine, tend to increase over time. This exacerbates the symptoms associated with the loss of acetylcholine in the brain (Peña-Bautista et al., 2020). Our findings are consistent with this evidence and determine a correlation between Choline and Alzheimer’s disease, although this relationship is not significant when time is considered. The application of Acetylcholinesterase inhibitors as a short-term, periodic, and symptom-relieving treatment in the initial stages of AD (Ferreira-Vieira et al., 2016; Rees & Brimijoin, 2003; Walczak-Nowicka Ł & Herbet, 2021), elucidates the failure of acetylcholine (one of the substantial choline-derived metabolites in AD pathology) as a biomarker for detecting the time progression AD.

Several studies have underscored the crucial role of Choline and choline-induced metabolites in AD. However, there is a discrepancy in the literature clarifying the exact alterations. Some studies documented lower Choline levels associated with AD (Judd et al., 2023; Mahajan et al., 2020), Lower levels of tryptophan betaine were observed predominantly in a sporadic subtype of AD (Novotny et al., 2023). Also, assessments were lower in people with cognitive impairments, specifically in females (Xu et al., 2021).

AD is positively correlated with high levels of oxidative stress. This oxidative stress can cause the oxidation of amino acids such as methionine, leading to detrimental effects on brain function (Chandran & Binninger, 2023).

Furthermore, other studies have suggested that changes in serum metabolites may precede the development of amyloid plaques and other pathological features of AD. This raises the intriguing possibility that alterations in metabolism could be involved in the early stage of AD pathogenesis and may represent a potential target for early intervention and prevention strategies (;“https://bmcbiochem.biomedcentral.com/articles/10.1186/1471-2091-13-21,” ; Mahapatra et al., 2023).

Though investigating amino acid levels, specifically (BCAA), including leucine, valine, and isoleucine, have gained popularity, the results are still under negotiation. Several studies emphasized the positive relationship between circulating BCAA and AD pathology (Ibáñez et al., 2012; Ikeuchi et al., 2022; Larsson & Markus, 2017; Morabito et al., 2014; Siddik et al., 2022). Nonetheless, some other studies’ outcomes spotted a negative correlation (Qian et al., 2023; Tynkkynen et al., 2018). Generally, lower serum valine levels were reported in neurodegenerative diseases in prior studies (González-Domínguez, García-Barrera, & Gómez-Ariza, 2015; Ibáñez et al., 2012; Jon B Toledo et al., 2017; Xiong et al., 2022). In a cross-sectional investigation using capillary electrophoresis-mass spectrometry constructed by Clara Albanez, lower levels of valine were found to have a stimulatory correlation in MCI rather than in AD subjects. On the other hand, a positive connection was determined to AD, suggesting valine as a biomarker for detecting the conversion of MCI to AD (Ibáñez et al., 2012). A more recent study conducted a targeted analysis of serum valine using ultra-performance liquid chromatography demonstrated conflicting results. The findings of this study postulate decreased levels of valine in AD rather than in MCI state with no significant predictive values identified to early differentiation of MCI and AD (Xiong et al., 2022). Aside from these studies, several works were conducted around the alternation of BCAAs as a risk factor for the occurrence of AD (Larsson & Markus, 2017; Siddik et al., 2022; Tynkkynen et al., 2018). However, Qian et al. reject the bi-directional correlation between these amino acids and AD, only accepting lower BCAA as an outcome ]in AD patients (Qian et al., 2023). Conflicting results may be linked to the requirement for more metabolomics-targeted research involving diverse and larger participant groups.

hypoxanthine is indicated to be tightly linked to acetylcholinesterase, which plays an indispensable role in AD pathology (Chen et al., 2020). Another investigation analyzed human brain samples and found that hypoxanthine levels differ depending on the brain region. For example, the frontal cortex exhibited the most alterations compared to the parietal and temporal cortex (Alonso-Andrés, Albasanz, Ferrer, & Martín, 2018). Amplified levels of hypoxanthine were reported in CSF (Kaddurah-Daouk et al., 2013) and plasma (Chouraki et al., 2017) samples, although one study found no changes in CSF (Degrell & Niklasson, 1988).

Previously, the majority of the investigations have clarified the role of the hypothalamic-pituitary-adrenal axis (HPA) in the pathology of AD. Activation of the HPA axis runs several hormonal pathways leading to cortisol secretion. These studies shed light on the association between chronic stress evoked by dysregulation in the HPA axis and cognitive impairment (Canet et al., 2019; Czech et al., 2012). However, our discoveries in line with other studies (Zheng, Tal, Yang, Middleton, & Udeh-Momoh, 2020) demonstrated no definite predictive significance for cortisol in the serum of AD patients. In healthy individuals without the presence of AD pathogenesis, amplified levels of morning awakening cortisol and decreased bedtime cortisol facilitate cognitive functions (Holleman et al., 2022).

Conversely, in people suffering from AD, measured awakening cortisol levels are correlated reversely with AD (Holleman et al., 2022; Zheng et al., 2020). Additionally, no considerable transitions were observed in afternoon and evening cortisol levels (Holleman et al., 2022). Inflammation followed by oxidative stress, hippocampal damage, and inappropriate regulation of the HPA system is hypothesized to be the underlying reason in AD patients (Juszczyk et al., 2021). Moreover, no substantial difference was spotted regarding mean cortisol levels in the mild and more severe forms of AD (Czech et al., 2012). Overall, the evidence suggests that cortisol does not have any helpful predictive capabilities in the advancement of the disease.

The pathology underlying AD disturbs metabolic pathways involving lipids due to inflammation and high oxidative state (Chew, Solomon, & Fonteh, 2020). Oleic acid (FA (18:1)) and linoleic acid (FA (18:2)) are both potential predictive biomarkers for AD pathogenesis. Our research analyzed these two products, which had higher F scores among metabolites of lipid pathways. Our analysis did not reveal any significant predictive value when considering the interaction of the two metabolites with time. However, when evaluating them independently, only linoleic acid exhibited notable predictive value. Most studies focus on fatty acid metabolites, particularly polyunsaturated fatty acids (PUFAs), as predictive biomarkers. The majority of these studies have concluded that both linoleic acid and oleic acid correlate well, and a decrease in levels is proposed in the case of AD pathogenesis (Cunnane et al., 2012; Shao et al., 2020; Snowden et al., 2017; Wang et al., 2012; Yin, 2023). Olive oil’s primary component, oleic acid, has been indicated to improve cognitive function in aging individuals (Sakurai, Shen, Shiraishi, Inamura, & Hisatsune, 2021; Santa-María et al., 2023; Valls-Pedret et al., 2015). While multiple studies have found decreased levels of oleic acid in patients with (AD) (Cunnane et al., 2012; Olazarán et al., 2015; Snowden et al., 2017; Yin, 2023), a report from R.González-Domínguez et al. revealed higher levels (González-Domínguez et al., 2015). Likewise, linoleic acid has been found to have a protective effect against neurodegenerative diseases, including AD (Kousparou, Fyrilla, Stephanou, & Patrikios, 2023), despite the pro-inflammatory nature of linoleic acid (Innes & Calder, 2018; Kousparou et al., 2023). Reported levels of linoleic acid were generally found to be lower during AD pathogenesis (Amick et al., 2022; Snowden et al., 2017; Wang et al., 2012; Yin, 2023). All aforementioned conflicting discoveries necessitate further specified research in this area. It is worth noting that assessments can vary depending on the targeted sources used, such as Erythrocyte fatty acids or Plasma phospholipid fatty acids (Olde Rikkert et al., 2014). Several substantial limitations need to be considered carefully. Even while all AD participants had access to metabolite data from the ADNI database, only a small portion of individuals had access to the PET scores at the designated time intervals. Consequently, the extreme conclusions may be impacted by the small sample size. Furthermore, we don’t appear to assess the impact of metabolites associated with gender. Furthermore, because the ADNI study is objective, it is challenging to assess the directionality of relationships and causal pathways and to manage for confounding. For example, environmental factors for each individual can have an impact on beta-amyloid and may be significant. Larger sample sizes should be used in future longitudinal research, and gender-associated metabolites must be included in AD prediction models. Future research can improve the generalizability of the results by including varied groups, ensuring a diversified cohort with varying genetic origins, lifestyles, and comorbidities. Further longitudinal research is necessary to examine the relationship between each selected metabolite item and AD.

It emphasizes the value of customized strategies and the importance of taking into account various metabolic pathways when uncovering their fundamental mechanisms and medical implications throughout various phases of cognitive decline.

This study provides evidence these seven metabolites affect the progression of Alzheimer’s disease by worsening brain hypometabolism. It also can affect the progression of the disease by impacting brain metabolism.

Our findings expand that Future longitudinal studies are required to be conducted to validate these seven metabolites and explore their potential in clinical practice.

## Conclusion

In conclusion, our results suggest a significant correlation between PET scores and seven metabolites such as DOPA, Met.SO, TrpBetaine, choline, Leu, Val and FA (18:2), which show promise as a potential alternative or complementary predictors (p < 0.05), without considering time interaction, Met.SO was particularly significant after adjusting time. Future longitudinal studies are required to investigate, the predictive value of these seven metabolites for AD progression.

## Figures and Tables

**Table 2:** Ten top metabolites predictive for amyloid PET scores

Metabolites	F-scores	P-value
Dopamine^1^	6.04	0.01
Methionine-Sulfoxide^1^	6.22	0.01
Tryptophan betaine^1^	5.61	0.02
Hypoxanthine^1^	7.45	0.01
Choline^1^	9.67	0.002
Cortisol^2^	4.55	0.03
FA(18:2)^2^	6.95	0.01
FA(18:1)^2^	6.11	0.01
Valine^2^	6.07	0.01
Leucine^2^	4.10	0.04

1: predictive metabolites for visit time 1

2: predictive metabolites for visit time 2

Metabolites predictive for visit time 1 are Dopamine, Methionine-Sulfoxide, Tryptophan betaine, Hypoxanthine, and Choline. Metabolites predictive for visit time 2 are Cortisol, FA(18:2), FA(18:1), Valine, and Leucine. Higher F-scores and lower P-values indicate a stronger predictive relationship with amyloid PET scores

**Table 3:** The predictive value of selected metabolites using a linear mixed effect model

Metabolite / Metabolite*time	Value	Std Error	t-value	p-value
Dopamine	0.008	0.004	2.294	0.024
Time* Dopamine	−0.001	0.003	−0.162	0.871
Methionine-Sulfoxide	0.015	0.005	2.821	0.006
Time* Methionine-Sulfoxide	−0.008	0.003	−2.413	0.017
Tryptophan betaine	0.009	0.004	2.236	0.027
Time* Tryptophan betaine	−0.003	0.004	−0.804	0.423
Hypoxanthine	0.003	0.004	0.722	0.472
Time* Hypoxanthine	0.001	0.005	0.195	0.846
Choline	0.008	0.003	2.596	0.011
Time* Choline	0.002	0.004	0.390	0.697
Leucine	0.010	0.003	3.383	0.001
Time* Leucine	−0.002	0.003	−0.615	0.540
Valine	0.009	0.003	2.989	0.003
Time* Valine	−0.002	0.003	−0.521	0.604
FA (18:1)	0.004	0.004	1.096	0.275
Time* FA (18:1)	0.002	0.004	0.438	0.662
FA (18:2)	0.007	0.003	2.009	0.047
Time* FA (18:2)	−0.003	0.003	−1.006	0.316
Cortisol	0.003	0.003	0.763	0.447
Time* Cortisol	0.002	0.004	0.359	0.721

**Table 4:** Result of linear mixed model in which selected metabolites were run as predictors of amyloid PET scores previously measured

Metabolites	*Value*	*Std.Error*	*t-value*	*p-value*	*Effect size*
Dopamine	0.01	0.003	2.34	0.021	0.002
Methionine-Sulfoxide	0.01	0.01	2.23	0.028	−0.005
Tryptophan betaine	0.01	0.004	2.1	0.038	0.004
Hypoxanthine	0.004	0.004	1	0.321	0.001
Choline	0.01	0.002	3.34	0.001	0.001>
Leucine	0.01	0.003	3.54	0.001	−0.002
Valine	0.01	0.003	3.06	0.003	−0.003
FA(18:1)	0.005	0.003	1.71	0.089	0.006
FA(18:2)	0.01	0.003	1.74	0.084	0.008
Cortisol	0.003	0.003	1.21	0.230	−0.001

## Data Availability

The datasets analyzed during the current study were obtained from the Alzheimer’s Disease Neuroimaging Initiative (ADNI) database. ADNI data are available to qualified investigators through the LONI Image and Data Archive, subject to registration, approval of a data access request, and compliance with the ADNI Data Use Agreement and publication policies. No new primary participant-level data were generated in this study. Because the data are governed by ADNI access and use policies, the authors are not permitted to redistribute individual-level data. Access to the data can be requested directly through the ADNI data access platform. Additional information regarding the analytic approach may be obtained from the corresponding author upon reasonable request.
